# Spinal cord morphology and PKD2L1^+^ cells distribution: effects of age, sex, and spinal segment in mice

**DOI:** 10.3389/fnana.2025.1652848

**Published:** 2025-10-24

**Authors:** Lugdivine Leblond, Jorge Ramirez-Franco, Caroline Michelle, Nicolas Wanaverbecq, Morgane Evin

**Affiliations:** ^1^Aix Marseille Univ, Univ Gustave Eiffel, LBA, Marseille, France; ^2^Aix Marseille Univ, CNRS, INT, Inst Neurosci Timone, Marseille, France

**Keywords:** spinal cord, central canal, mouse model, morphometry, confocal imaging

## Abstract

**Introduction:**

Morphometrical studies of the mouse spinal cord are often limited to one age or sex, restricting our understanding of anatomical variability. This study provides a detailed analysis of the spinal cord in mice, examining the effects of age, sex, and spinal region, along with the distribution of PKD2L1-positive (PKD2L1^+^) cells along the rostro-caudal axis.

**Methods:**

Using 811 transverse sections from a total of 18 3- and 8-week-old mice, DAPI immunofluorescence and confocal imaging, 14 dimensions of gray matter (GM), white matter (WM), and the central canal (CC) were assessed using landmarks positioning and segmentation methods.

**Results:**

Age was the most influential factor: between 3- and 8- weeks-old, the spinal cord showed reduced rostro-caudal length (*p* = 2.49e-04), smaller ventral GM horns (*p* < 0.005), deeper ventral commissures (*p* = 5.58e-13), and an increase in CC area (from 1925.58 ± 630.16 μm^2^ to 2199.50 ± 569.44 μm^2^). Looking at sex-related differences, females showed higher variability across several parameters, with subtle differences in GM organization (*p* < 0.05) and CC morphology (mean area = 2146.39 ± 632.91 μm^2^ in females vs. 1998.36 ± 589.85 μm^2^ in males). Along the rostro-caudal axis, WM size, as well as GM dorsal and ventral horn dimensions, differed significantly across spinal segments (*p* < 0.005). CC position also shifted dorsally in cervical and lumbar regions depending on age and sex (*p* < 0.005). PKD2L1^+^ cells were mainly clustered near the CC, with over 46% located proximally. The highest densities (>300 cells/segment) were found in lumbar and lower thoracic regions.

**Discussion:**

These results indicate progressive structural changes during development, including reorganization of cells and CC architecture stabilization. The distribution of PKD2L1^+^ cells is consistent with their proposed role as cerebrospinal fluid-contacting neurons potentially involved in sensing fluid composition and modulating locomotor control. Their increased presence in caudal segments suggests functional specialization in different spinal regions.

**Conclusion:**

This work provides detailed, segment-specific anatomical data crucial for developing accurate and physiological numerical models. Adding age and sex differences emphasizes the need to reflect biological variability in simulations. Additionally, the mapping of PKD2L1^+^ neurons offers valuable insight into their spatial organization and potential involvement in sensory processing, locomotor function, and neurological or developmental disorders.

## 1 Introduction

As a crucial component of the central nervous system, the spinal cord not only transmits signals between the brain and the body’s muscles and organs but also processes sensory inputs from peripheral receptors and contributes in sensing bodily states to regulate and ensure homeostasis. Due to their genetical similarities with Humans, mice are the experimental model predominantly used to investigate spinal cord organization in physiological and pathological conditions (Breschi et al., 2017; Monaco et al., 2015), as well as in spinal cord injury research (Fournely et al., 2020; Reinhardt et al., 2020; Reshamwala et al., 2020). It contributes to better understand spinal cord diseases (Stephenson and Amor, 2017; Xin et al., 2024), to assess therapeutic strategies for axonal regeneration (Shu et al., 2024), and functional recovery (Kong et al., 2021). Despite the extensive use of this model, quantitative anatomical data integrating multiple structural features across biological variables remain limited.

Most anatomical studies focus on the spinal cord internal structures, such as the White (WM) and the Gray Matter (GM) morphology or the central canal (CC) characteristics. The WM is composed of ascending and descending axonal tracts that, respectively, transmit sensory inputs from peripheral regions to the brain, and motor commands from the brain to the muscles and organs (Schröder et al., 2020). The GM, organized in a bilateral hornlike configuration, contains neuronal cell bodies and is functionally subdivided into 10 *laminae* based on cytoarchitectural criteria (Watson et al., 2009). The dorsal horns typically process somatosensory inputs while the ventral horns contain motor neurons, and the lateral horns house neurons involved in autonomic control (Mercadante and Tadi, 2025).

Located at the center of the spinal cord, the central canal (CC), a narrow tube and extension of the cerebral ventricular system, is present along the whole spinal axis and filled with cerebrospinal fluid (CSF) (Alfaro-Cervello et al., 2014; Cañizares et al., 2020). The region around the CC comprises of an heterogenous cellular population with the ependymal cell monolayer forming its wall, glial and stem/progenitor cells (see also below). This region was shown to exhibit reparatory capabilities and to be implicated in regenerative processes following injury (Lacroix et al., 2014; Liu et al., 2021; Rodriguez-Jimenez et al., 2023; Yue et al., 2024). The CC is suggested to have collapsed below the cervical region in adult Humans (Torrillas de la Cal et al., 2021) and age-related morphological changes and CC dilatation associated with syringomyelia (Chang, 2021; Petit-Lacour et al., 2000) were reported. However, recent studies have shown that the CC might be preserved along the whole spinal cords (Lefevre et al., 2025; Saker et al., 2016; Storer et al., 1998; Yasui et al., 1999). One main reason for such discrepancy in the observation may be of technical nature with the a CC collapse influenced by histological slicing artifacts, raising questions about the accuracy of these observations (Solsberg et al., 1990). CC dilatation has also been associated with pathologies such as syringomyelia (Chang, 2021; Petit-Lacour et al., 2000). Though the functional role of the CC is well studied, its morphological and anatomical variability remains poorly characterized in rodents and further anatomical analysis are necessary.

Interestingly, a unique population is observed around the CC, the cerebrospinal fluid-contacting neurons (CSF-cNs). They are observed in all vertebrates within or beneath the ependymal cell layer, exhibit a characteristic morphology with a dendrite extending into the canal and ending with a ciliated protrusion (Jurčić et al., 2021). Further, they selectively express the PKD2L1 channel, a member of the Transient Receptor Potential (TRP) with sensory properties (Orts-Del’Immagine et al., 2016, 2012; Sternberg et al., 2018). These neurons were shown to be tightly coupled with ependymal cells (Stoeckel et al., 2003) and to modulate their development (Di Bella et al., 2019) and proliferation (Yue et al., 2024). These reports therefore suggest that CSF-cNs could participate in shaping the CC and the surrounding region. At a functional level, they were shown to detect cerebrospinal fluid flow (Jalalvand et al., 2016; Knafo and Wyart, 2018; Orts-Del’Immagine et al., 2020), composition (Huang et al., 2006; Orts-Del’Immagine et al., 2016, 2012) and spinal cord bending (Böhm et al., 2016; Sternberg et al., 2018). Finaly, more recently, studies in the mouse indicated that CSF-cNs, in particular in the lumbar segments, would participate in the modulation of motor control (Gerstmann et al., 2022; Knafo and Wyart, 2018; Nakamura et al., 2023; Orts-Del’Immagine et al., 2020). CSF-cNs therefore would represent key actors for the organization of the CC region however, their precise distribution patterns and how these might vary across developmental or anatomical contexts remain unclear.

Most anatomical studies focus on isolated aspects, including WM and GM as well as GM *laminae* organization (Watson et al., 2009), and have mapped neuronal networks to elucidate intercellular connectivity (Bukreeva et al., 2017) and spinal neurons positions in a 3D reference atlas (Fiederling et al., 2021). However, they lack providing quantitative description of spinal cord morphology and involve a limited number of animals, often failing to take into account inter-individual variability linked to sex, age, or body weight. Furthermore, atlases rely on histologically experiments and results often limited to one age and sex and requiring dehydration protocols that prevent degradation but induce tissue shrinkage and hardening (Idziak et al., 2023; Nicolle and Palierne, 2010; Troiano et al., 2009). *In-vivo* MRI-based approaches offer a potential alternative by providing realistic morphological analysis. However, even in ultra-high magnetic fields (≥9.4 T), the in-plane resolution achieved in most MRI protocols ranges between 60 and 100 μm, with slice thicknesses typically ≥200 μm. This resolution is constrained by signal-to-noise ratio, long acquisition time and animal motion *in vivo*. Most published studies using high-resolution MRI in the mouse spinal cord do not report direct visualization of the CC as only WM and GM boundaries are detected (Blomster et al., 2013; Gatto et al., 2018; Saito, 2006). Some studies have integrated MRI images analysis to determine vertebral landmarks useful in surgical planning (Harrison et al., 2013).

Altogether, the reported studies lack quantitative descriptions of spinal cord morphology and involve a limited number of animals, often without analysis of inter-individual variability linked to sex, age, or body weight. Integrated a quantitative analysis of spinal cord morphology along the whole axis and considering PKD2L1^+^ cells distribution remains crucial to better understand spinal cord organization and evolution with ages and pathological conditions.

The present study aims at contributing to fill this gap and quantitatively characterizing the morphology of WM, GM, the CC, as well as the distribution of PKD2L1^+^ cells across spinal regions in male and female mice of different ages. Immunohistofluorescence staining and confocal microscopy are used to generate a multi-parameter dataset. This approach addresses the current lack of integrated morphological analyses that consider biological variability, aiming at improving the anatomical basis for interpreting functional and pathological studies of the spinal cord.

## 2 Methods

### 2.1 Experimental model

**Figure 1:**
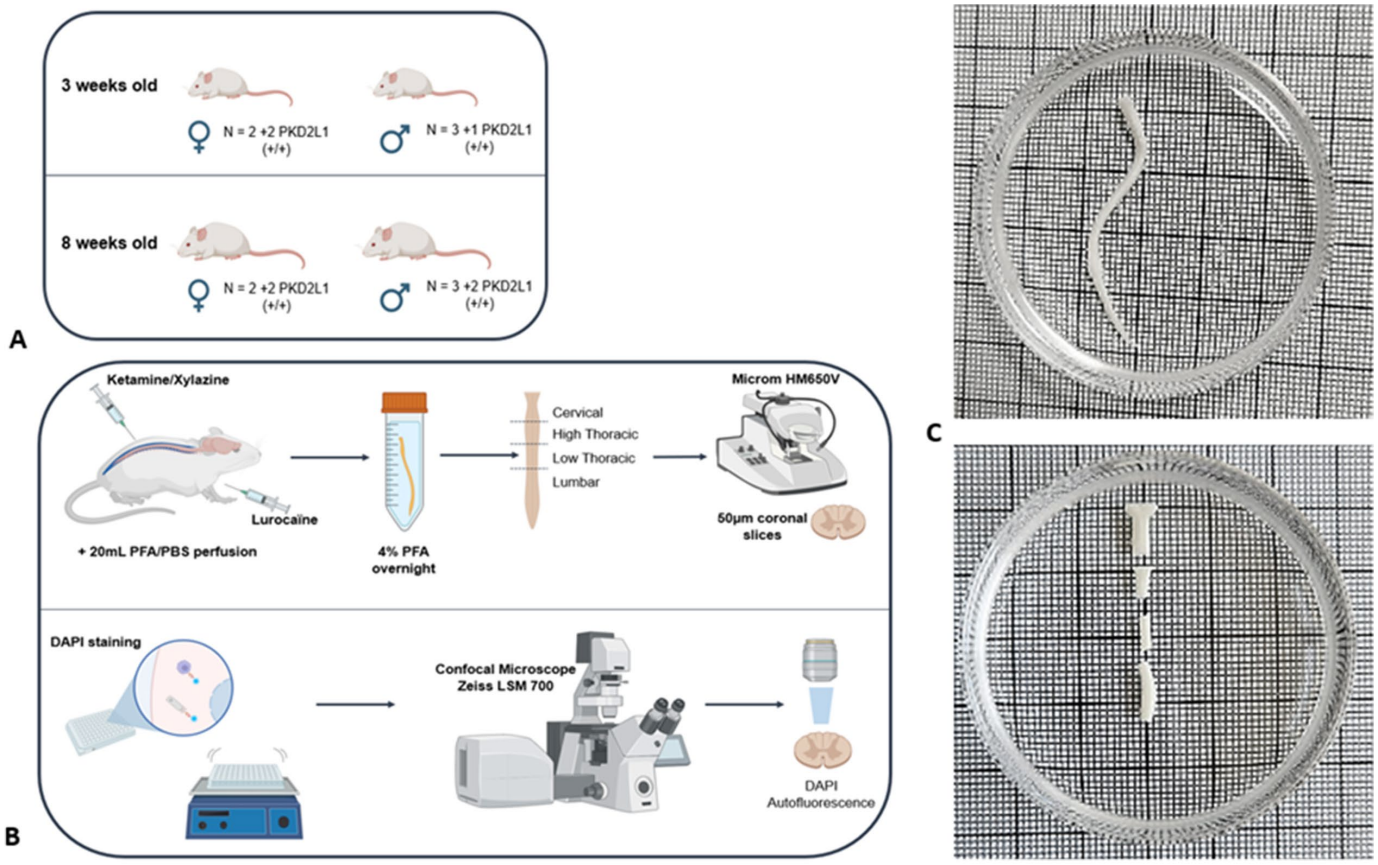
Samples preparation methodology. **(A)** Mice cohorts used for the study **(B)** Immunohistochemistry protocol **(C)** Spinal cord division into cervical, high thoracic, low thoracic and lumbar segment.

All animal procedures were following ethical regulations for animal use, and all experiments were conducted in conformity with the rules set by the EC Council Directive (2010/63/UE) and the French “Direction Départementale de la Protection des Populations des Bouches-du-Rhône (DDPP13)” (Project License Nr: APAFIS 44331,2023071917567777 & 33336, 2021101819022439. WN and License for the Use of Transgenic Animal Models Nr: DUO-5214). Protocols used agree with the rules set by the Comité d’Ethique de Marseille (CE71), our local Committee for Animal Care and Research. All animals were housed at constant temperature (21 °C), in an enriched environment, under a standard 12 h light-12 h dark cycle, with food (pellet AO4, UAR, Villemoisson-sur-Orge, France) and water provided ad libitum. Every precaution was taken to reduce to the minimal the number of animals used and minimize animal stress during housing and prior to experiment. Experiments were carried out on a total of 18 mice, divided equally between two age groups: 3-weeks-old (3wo) (*n* = 9): 3 wild-type females, 2 wild-type males, 2 Pkd2l1-Cre::tdTomato females, and 2 Pkd2l1-Cre::tdTomato males. 8-weeks-old (8wo) (*n* = 9): 2 wild-type females, 2 wild-type males, 2 Pkd2l1-Cre::tdTomato females, and 3 Pkd2l1-Cre::tdTomato males (Supplementary Table 1). PKD2L1-Cre (Pkd2l1^tm1(cre)^; MGI ID: 6451758; a generous gift Emily Leman) were cross-breed with flex-tdTomato mice (Gt (ROSA)26^Sortm14 (CAG-tdTomato)Hze^, The Jackson Laboratory, MGI ID: 3809524; RRID:IMSR_JAX:007914) to selectively express the tdTomato fluorescent protein in the neuronal population of interest. All Wild-type mice used were from the C57BL/6 J strain (Charles River, MGI ID: 3028467). This design allowed for balanced sex representation within each age group and ensured the collection of a large number of anatomical sections per spinal segment, resulting in 811 analyzed transverse sections in total. This dataset enables reliable morphological comparisons, despite a modest number of animals (Figure 1A and see Supplementary Table 1). This dataset enables reliable morphological comparisons, despite a modest number of animals. The number of mice used was chosen in agreement with the 3R principles (Replacement, Reduction, Refinement). The 3-weeks-old mice represent a developmental stage shortly after weaning and prior to the full maturation of spinal cord architecture, while the 8-weeks-old group corresponds to young adult mice with more stabilized structures. This age selection was designed to capture morphometric differences associated with postnatal developmental changes.

### 2.2 Experimental procedure

Animals were randomly selected from their respective litters within each group. Thirty minutes before the procedure, mice were injected with Metacam (5 mg/kg). Anesthesia and analgesia were induced by intraperitoneal injection of a mixture of Ketamine (100 mg/kg) and Xylazine (15 mg/kg). Paw-pinch reflex was tested to ensure animals were deeply anesthetized. A subcutaneous injection of Lurocaine (5 mg/kg), a local analgesic, was performed on the sites of incision prior to surgery. Animals were then intracardially perfused with 20 mL of phosphate buffer solution (PBS) at 37 °C followed with 20 mL of ice-cold paraformaldehyde (PFA) in PBS (4% PFA-PBS). Vertebrae, nerve roots and meninges were carefully removed. Spinal cord tissue was then collected and post-fixed 24 h in 4% PFA-PBS solution at 4 °C. The spinal cord was then divided into four spinal segments (cervical, high thoracic, low thoracic and lumbar) (Figure 1B).

Segments were sliced using a vibratome (Microm HM650V) with a cutting speed of 0.8 mm/s at a thickness of 50 μm, collecting 4 sections each time, then leaving a 400 μm gap before repeating the procedure. Floating sections were collected into 24-well plates containing DAPI solution (1.5 μg/mL) in PBS. After washing 2 times (15 min/each) in PBS, sections were mounted on a microscope slide. 22 ± 4 sections were retrieved per segments for a total number of 811 (401 and 410 for the 3- and 8-weeks-old mice respectively).

### 2.3 Image acquisition

**Figure 2:**
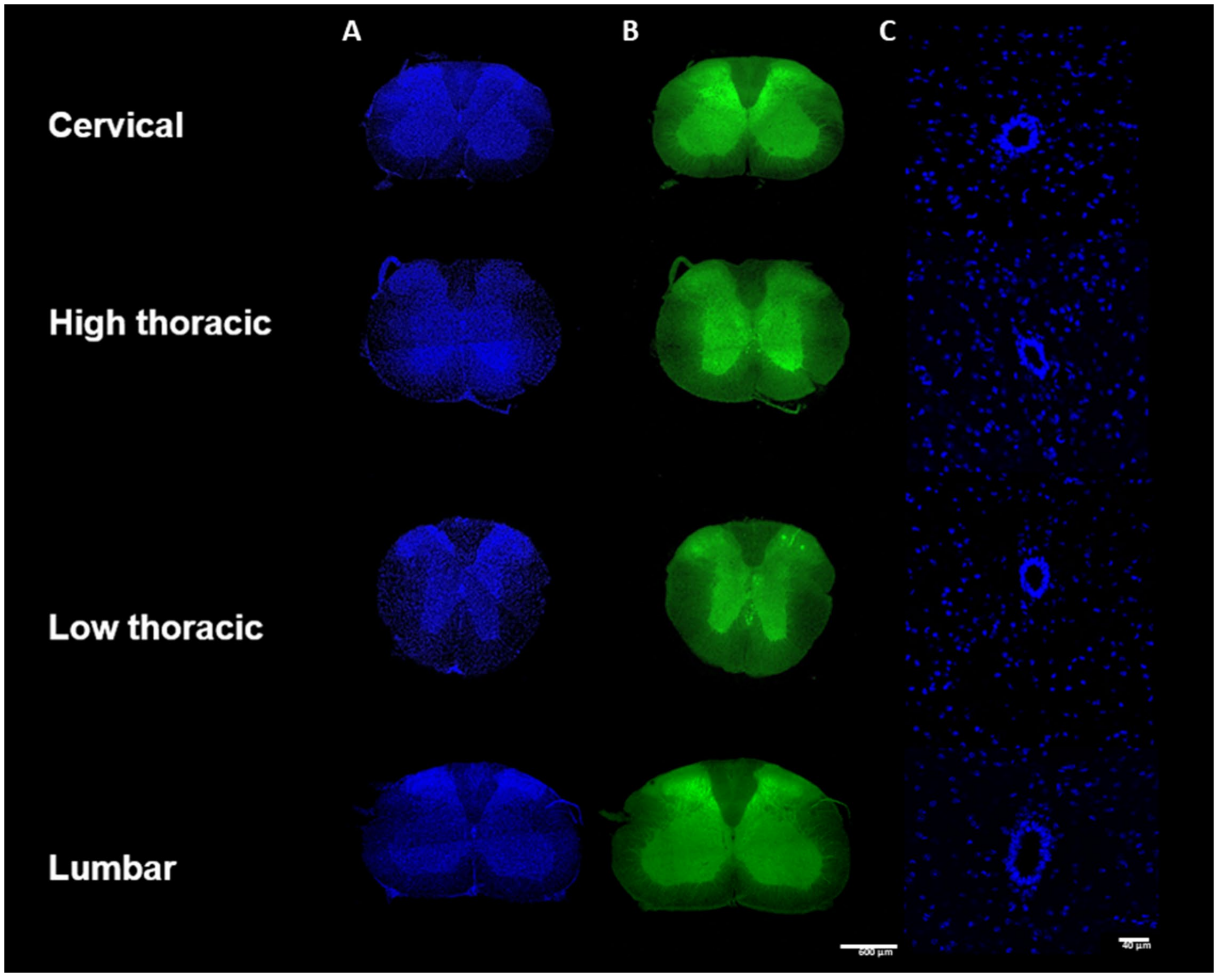
Samples preparation methodology– **(A)** Immunohistochemistry protocol **(B)** Spinal cord division into cervical, high thoracic, low thoracic and lumbar segment.

**Figure 3:**
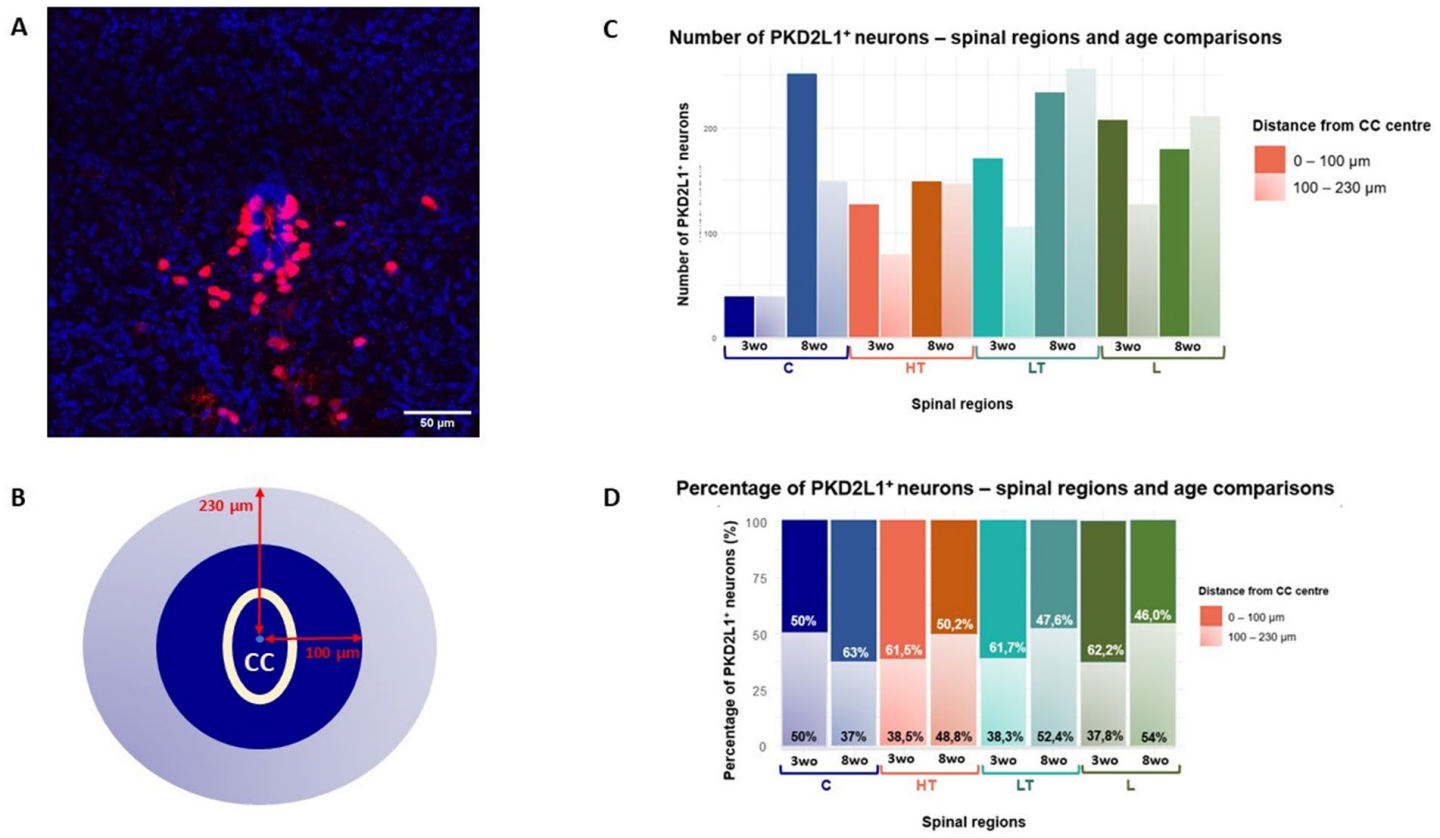
**(A)** Confocal microscopy image showing the CC (in blue) and the PKD2L1^+^ cells around were detected with the expression of tdTomato (red). Scale bar, 50 μm. **(B)** Space around the CC was divided in two areas at a distance of 100 μm (green) and 230 μm (orange) around the CC center. **(C)** Quantitative analysis of cells distribution and **(D)** their percentage according to their distance from the CC center across spinal segments and age groups.

The spinal cord sections were imaged using confocal scanning microscopy (LSM 700, Carl Zeiss, Germany). 811 images of the whole spinal cord were first acquired with a x10 magnification with a x0.5 digital zoom (image size 1279.1×1279.1 μm, pixel size 1.25 μm, voxel depth 5.35 μm, optical thickness 5.36 μm, 2×2 images/mosaic, stack depth 25-37 μm). Two channels were sequentially acquired: 405 nm-blue for DAPI (Figure 2A) and 488 nm-green for the auto fluorescence (Figure 2B). 811 images of the CC were then imaged with a x20 magnification with a x1.0 digital zoom (image size 319.8×319.8 μm, pixel size 0.31 μm, voxel depth 0.85 μm, optical thickness 0.86 μm, stack depth 25-37 μm) (Figure 2C). One channel was acquired for the DAPI. Additionally, for the Pkd2l1-Cre::tdTomato mice, the 555 nm-red channel specific to the td Tomato fluorescent protein was acquired, respectively, for the two stacks of images (Figure 3A).

### 2.4 Image analysis

Image analysis was performed under the same protocol and conditions, regardless of the animal’s age, sex, or genotype during segmentation and measurement. Images were prepared using Fiji (ImageJ 2.14) (Schindelin et al., 2012). Images’ contrast and brightness were adjusted in order to ensure better visualization and the sections were reoriented so that the horns follow the ventro-dorsal axis. A maximum intensity *Z*-projection was applied on images of the whole spinal cord. Obtained images were analyzed using a home-made Python code (Python version 3.11.7, Python Software Foundation, Python Language Reference) and a Matlab code (MATLAB Release 2023, The MathWorks, Inc., Natick, Massachusetts, United States) available upon reasonable request.

#### 2.4.1 White and gray matters

**Figure 4:**
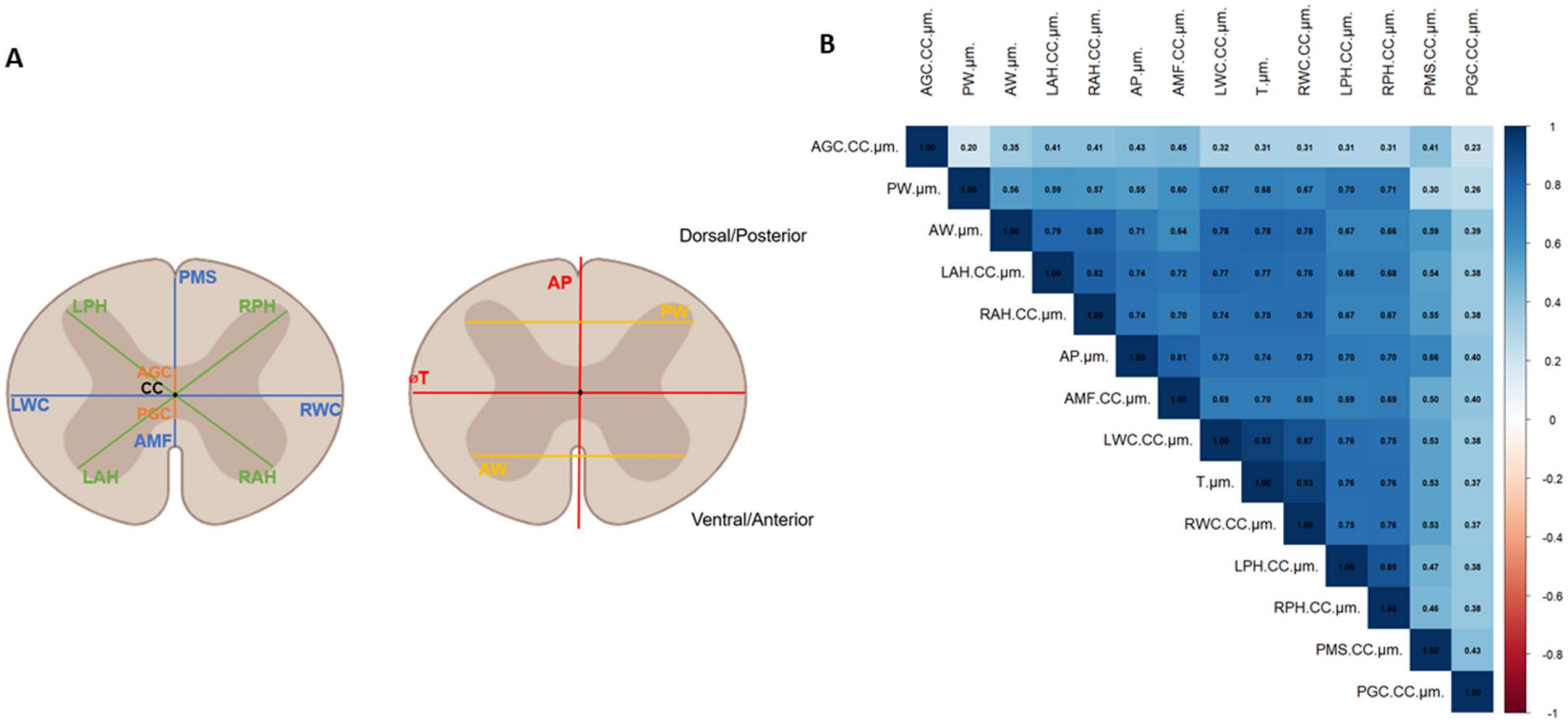
**(A)** Morphological parameters measured on confocal images of spinal cord sections: AP, ⌀T, VWC, DMS for the White Matter; LWM, MWM, AW, PW, LVH, RVH, LDH, RDH for the Gray Matter and VGC and DGC for the central canal. **(B)** Correlation matrix showing linear relationship between all morphological parameters. The correlation coefficient indicates the degree of correlation (0.1–0.3: low correlation, 0.3–0.5: moderate correlation, 0.5–0.7: strong correlation, 0.7–0.9: highly strong correlation, 1: perfect correlation).

Morphometrical analysis was carried out by making several measurements on spinal cord’s slices. Some measurements were derived from the morphological definition in Human (Fradet et al., 2014) as ⌀T = spinal cord transverse diameter, ⌀AP = spinal cord anteroposterior diameter, AW = GM anterior horns, PW = GM posterior horns. Additionally, 10 measurements were computed taking the CC as a reference: VGC = ventral gray commissure, DGC = dorsal gray commissure, LDH = left dorsal horn, RDH = right dorsal horn, LVH = left ventral horn, RDH = right dorsal horn, VWC = ventral white commissure, DMS = dorsal median sulcus, LWM = lateral WM, MWM = medial white matter. The landmarks were identified and placed manually on the GM and WM (Figure 4A).

#### 2.4.2 Central canal

**Figure 5:**
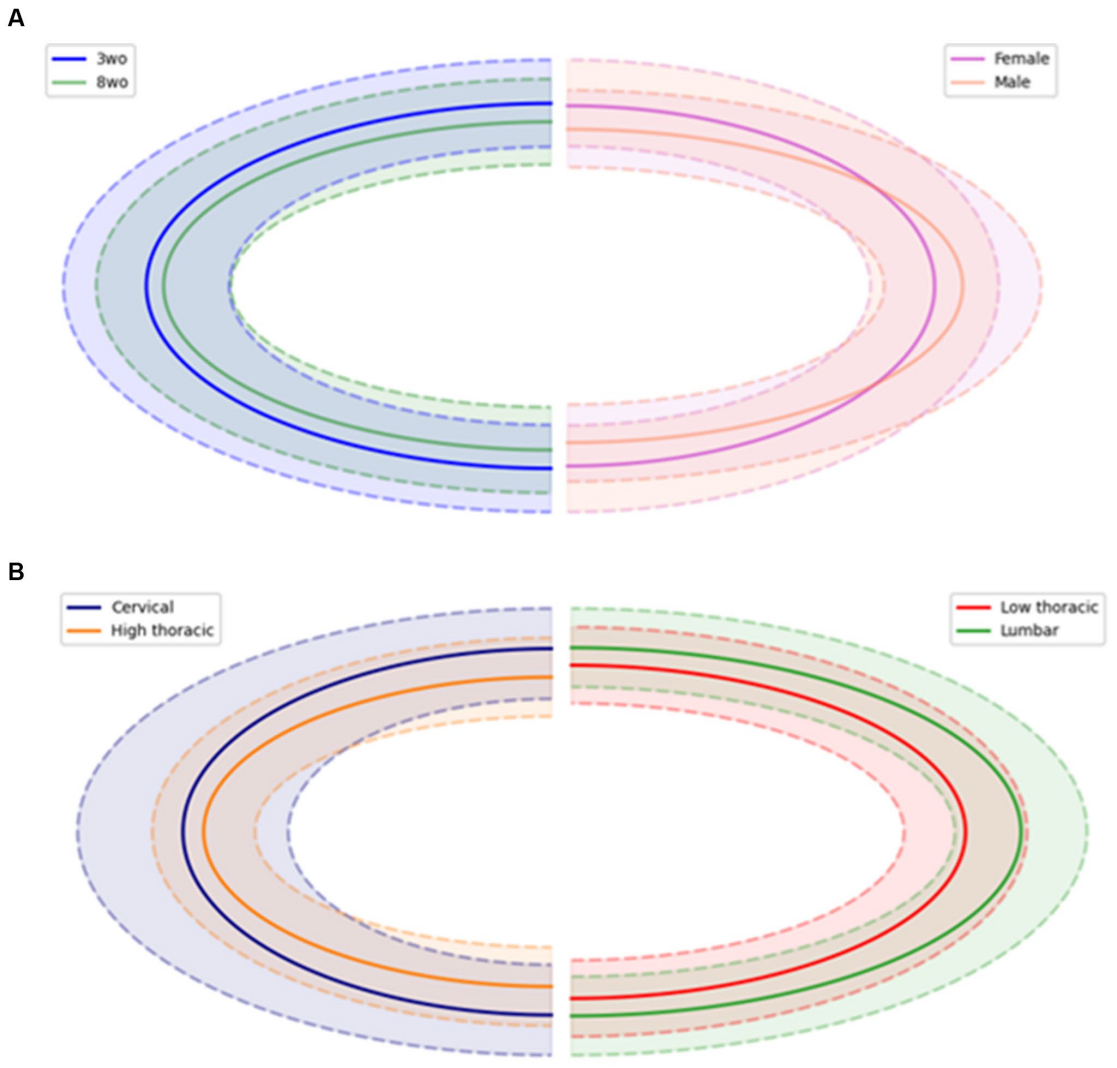
Central canal mean shape (in solid line) comparison between **(A)** age and sex, **(B)** spinal regions. Dotted lines represent the standard deviation of the mean shape.

A custom Python script was developed to identify the outline of the CC. For each spinal cord slice, a binary mask was generated using Otsu’s thresholding method. Connected components were filtered based on a minimum size threshold to isolate the relevant CC structures. Unsuccessful segmentations, in which the CC contour was not closed, were excluded from the study. If the segmentations were successful, contours of the CC were extracted, and an ellipse was fitted to the region to extract geometric parameters including the center coordinates, smallest and largest diameters, orientation, and ellipse area. Using these parameters, CC’s mean shapes were plotted for the different ages, sexes and spinal segments (Figure 5).

#### 2.4.3 PKD2L1^+^ cells

In Pkd2l1-Cre::tdTomato mice, tdTomato expression labels PKD2L1^+^ cells, primarily corresponding to CSF-cNs located near the CC. However, some tdTomato-labeled cells are observed at greater radial distances and may not be CSF-cNs *per se*, in particular for animal older than 6 weeks-old. For this reason, the potential heterogeneity of the distal population was acknowledged and PKD2L1^+^ cells were grouped in proximal cells representing CSF-cNs and distal ones whose nature is less documented. We made this distinction throughout the manuscript, were cautious in our interpretation regarding these two populations and mainly focused on cells present around the CC.

Confocal images of the PKD2L1^+^ cells were processed using a home-made Python code enabling segmentation and counting. Histogram normalization (using min-max scaling) and contrast enhancement (using standard histogram equalization based on cumulative distribution function) were applied independently to each slice in the image stack. A global intensity threshold, set at 95% of the maximum intensity, was used to generate binary masks. Objects’ volume smallest than 498 μm3 (6,000 voxels) were removed. The remaining features after deletion of small objects, representing the neurons, were counted and located by their centroid coordinates. The coordinates of the CC center for each slice were imported from the previous analysis to compute the distance between this reference and the neurons’ centroid.

### 2.5 Statistics

Reproducibility of the landmark’s placement was assessed by repeating the placements 3 times on three spinal segments. The coefficient of variations (COV) was computed for each measured lengths and the acceptable threshold was set to COV < 5%. Significant ratio and dimensions were computed using the measured lengths. Linear models were fitted to assess the influence of genotype on morphological parameters, with genotype, age, and sex included as fixed effects. We tested whether the data on spinal cord structure obtained from wild-type and Pkd2l1-Cre::tdTomato mice were statistically significantly different using ANOVA test (Supplementary Table 2). Since no statistical difference was observed, we merged the data to comply with the 3Rs ethical rules and to limit the number of animals used in our study. *p*-values were reported as follows: 0.01 < *p* < 0.05 *, 0.001 < *p* < 0.01 **, and *p* < 0.001 ***. Assumptions of normality and homogeneity of variance were verified prior to analysis to ensure model validity. The values can now be seen the supplementary data (Supplementary Table 2)”.

In total, 811 sections from 18 animals were analyzed. Sections were treated as statistical units but are nested within animals, which preserves inter-animal variability and avoids pseudo replication. Given the number of morphometric parameters tested, the risk of inflated significance was acknowledged. As the study is exploratory, no formal multiplicity correction was applied, and emphasis was placed on consistent effects across related parameters. Statistical analysis was performed using R (R Core Team (2021). R: A language and environment for statistical computing. R Foundation for Statistical Computing, Vienna, Austria. URL https://www.R-project.org/). N, the number of sections, was 401 and 410 for the 3- and 8-weeks-old groups and 413 and 398 for the female and male groups, respectively. All results are presented as mean ± sd [minimum value, maximum value]. An ANOVA test was performed to assess the statistical difference between the Wild-Type and PKD2L1^+^ mice. Significant *p* value was set to <0.05. Normality distribution of the data was determine using Shapiro–Wilk test. Depending on the result, a Student t-test or a Wilcoxon Mann–Whitney test was used to assess statistical differences between sex, age and spinal regions. To further evaluate the effect of spinal regions, a MANOVA test was performed on the parameters that previously showed significant differences after performing an ANOVA test. Prior to MANOVA, assumptions of multivariate normality were evaluated using the Shapiro–Wilk test. Correlation between the lengths was determine using the Kendall method. Results were visualized in a correlation matrix (Figure 4B).

## 3 Results

### 3.1 Reproducibility of the landmarks positioning protocol

The repeatability analysis showed an acceptable level of reproducibility, with a coefficient of variation below the 5% threshold, which supports the reliability of our measurements. Highest variability was nonetheless found for the VGC and DGC measurements (Table 1).

**Table 1:** Repeatability of landmarks positioning.

Spinal regions	High thoracic		Low thoracic		Lumbar	
*N*	24		23		30	
COV (%)	Mean	Std	Mean	Std	Mean	Std
AP	1	0.6	0.9	0.5	1	0.6
T	0.8	0.5	0.7	0.4	0.7	0.3
VWC	1.4	0.9	1.4	0.7	1.3	0.7
DMS	1.8	1.2	1.7	1	3.5	1.7
LWM	1.1	0.8	1	0.5	1	0.6
MWM	1.2	0.7	0.9	0.5	1.1	0.7
AW	1.1	0.8	0.9	0.5	0.9	0.5
PW	1.1	0.6	1.4	0.9	1	0.6
VGC	3.8	2.2	5.2	4.1	2.9	1.7
DGC	4.9	2.3	5.4	3.1	6	3
LVH	0.8	0.4	1	0.7	0.9	0.5
RVH	1.1	1.2	1.4	0.7	1	0.6
LDH	1.9	1.1	1.5	1	1.4	0.8
RDH	1.5	0.8	1.7	1.2	1.8	0.9

### 3.2 Morphological results by age and sex

#### 3.2.1 White and gray matter

For the 4 segments of interest, results were obtained by pooling both Wild-type and PKD2L1^+^ mice, as no significant differences were found between the two genotypes (Supplementary Table 2). This allows the analysis of 811 slices (202 and 199 slices for the 3wo females and males, respectively, and 211 and 199 slices for the 8wo females and males respectively) (Supplementary Table 1). The AP distances were significantly influenced by both sex (*p* = 1.93e-05) and age (*p* = 2.49e-04), with the highest mean values observed in 3 weeks-old females (878.01 ± 298.40 μm) compared to 3 weeks-old males (673.31 ± 96.89 μm) (Table 2). These differences may reflect sex-specific developmental trajectories of spinal cord growth, potentially influenced by hormonal or genetic factors. Similar results were observed for the transverse diameter ⌀T which was significantly affected by sex (*p* = 2.56e-08), though not by age (*p* = 0.12). The VWC distance showed no significant variation with sex or age. In contrast, DMS measurements were significantly affected by both sex and age (*p* < 0.001), with the largest mean value also observed in 3-weeks-old females (402.89 ± 130.90 μm). The AP-⌀T ratio remained stable across groups, with no significant influence of sex (*p* = 0.06) or age (*p* = 0.15).

**Table 2:** Morphological parameters values and comparisons between age and sex.

Lengths (μm)		Female_3wo	Male_3wo	Female_8wo	Male_8wo	*p*-value sex	*p*-value age
White Matter	AP	878.01 ± 298.40 [418.46, 1374.36]	673.31 ± 96.89 [249.56, 1168.98]	735.71 ± 85.84 [558.59, 1017.34]	765.44 ± 94.43 [564.34, 1005.82]	1.93e-05***	2.49e-04***
	T	1159.22 ± 393.56 [473.75, 1792.45]	915.50 ± 192.21 [448.85, 1621.64]	1024.56 ± 182.79, [731.61, 1707.31]	1013.55 ± 188.18 [632.84, 1400.51]	2.56e-08***	0.12
	VWC	422.39 ± 156.81 [136.3, 706.39]	332.35 ± 47.77 [232.37, 491.4]	335.62 ± 59.79 [243.8, 919.45]	358.09 ± 48.54 [249.56, 482.98]	0.12	0.052^●^
	DMS	402.89 ± 130.90 [190.07, 880.28]	291.71 ± 59.43 [120.94, 623.87]	358.61 ± 63.71 [251.46, 919.45]	362.48 ± 53.98 [264.95, 504.89]	1.10e-11***	2.941e-14***
Gray Matter	LWM	579.94 ± 194.88 [232.41, 921.89]	460.25 ± 97.82 [314.59, 838.76]	515.71 ± 97.23 [347.53, 919.45]	506.61 ± 95.44 [309.32, 717.28]	1.38e-08***	0.17
	MWM	580.02 ± 201.26 [241.44, 1067.43]	456.53 ± 96.47 [296.3, 782.94]	513.33 ± 96.45 [354.83, 919.45]	507.02 ± 94.87 [318.25, 695.34]	3.66e-08***	0.099
	AW	987.45 ± 387.36 [371.29, 1627.86]	734.75 ± 151.65 [433.58, 1408.48]	820.01 ± 192.14 [552.39, 1538.08]	787.45 ± 136.96 [524.96, 1237.22]	2.24e-07***	0.71
	PW	680.88 ± 250.35 [210.37, 1328.85]	621.53 ± 221.43 [319.08, 1086.29]	586.47 ± 175.53 [338.39, 972.22]	617.61 ± 210.97 [245.12, 1043.4]	0.71	0.0088**
	LVH	543.62 ± 209.04 [234.4, 885.4]	104.41 ± 51.24 [5.98, 307.64]	97.87 ± 64.81 [5.76, 919.45]	93.89 ± 38.81 [14.71, 301.92]	1.71e-04***	9.017e-08***
	RVH	547.09 ± 211.87 [226.76, 874.99]	64.58 ± 27.35 [17.37, 226.45]	63.17 ± 64.04 [5.3, 919.45]	72.90 ± 28.10 [19.96, 256.81]	0.068^●^	2.17e-04***
	LDH	400.23 ± 123.78 [219.29, 831.04]	393.65 ± 65.14 [285.97, 651.36]	418.66 ± 80.39 [311.19, 919.45]	414.52 ± 63.16 [267.5, 613.37]	2.27e-07***	0.26
	RDH	411.99 ± 129.02 [226.39, 942.96]	398.35 ± 64.56 [289.04, 673.08]	423.70 ± 80.15 [309.47, 919.45]	427.57 ± 66.86 [294.9, 655.21]	5.85e-06***	0.62
Central Canal	VGC	145.27 ± 82.10 [8.5, 337.85]	321.15 ± 85.23 [159.31, 606.11]	327.48 ± 74.87 [234.37, 919.45]	339.60 ± 75.61 [190.37, 563.45]	8.10e-05***	0.10
	DGC	105.05 ± 64.90 [5.3, 508.73]	324.90 ± 82.00 [206.65, 552.26]	329.88 ± 74.94 [233.93, 919.45]	339.51 ± 76.98 [101.72, 519.57]	1.2e-05***	0.015*
Ratio							
White Matter	AP/T	0.77 ± 0.08 [0.57, 0.96]	0.75 ± 0.09 [0.53, 1.09]	0.73 ± 0.09 [0.47, 1]	0.77 ± 0.10 [0.57, 1.08]	0.06^●^	0.15
	Commiss_size	455.62 ± 148.24 [257.13, 979.11]	342.52 ± 54.66 [222.66, 677.58]	400.10 ± 148.24 [257.13, 979.11]	407.35 ± 53.13 [314.78, 539.39]	2.13e-08***	5.58e-13***
Gray Matter	AW/PW	1.47 ± 0.29 [0.78, 1.94]	1.29 ± 0.38 [0.66, 1.97]	1.48 ± 0.40 [0.82, 2.82]	1.38 ± 0.35 [0.76, 2.73]	2.03e-07***	0.32
	RVH/RDH	1.31 ± 0.25 [0.75, 1.79]	1.27 ± 0.23 [0.73, 2.07]	1.30 ± 0.17 [0.84, 1.97]	1.30 ± 0.30 [0.65, 4.42]	0.03*	0.14
	LVH/LDH	1.34 ± 0.23 [0.66, 1.74]	1.27 ± 0.23 [0.83, 2.26]	1.30 ± 0.17 [0.80, 2.06]	1.25 ± 0.18 [0.50, 1.95]	7.63e-06***	0.0001***
Central Canal	VGC + DGC	250.32 ± 108.51 [63.62, 665.18]	168.99 ± 61.49 [75.06, 474.44]	161.04 ± 122.76 [68.67, 1838.9]	166.78 ± 149.08 [92.3, 462.01]	1.72e-05***	1.22e-11***

Data are classed according to mice age and sex, and to parameters location (White matter, Gray matter or Central Canal) – Mean ± standard deviation [minimum value, maximum value] – 0.05 < *p*-value < 0.07: ^●^, 0.01 < *p*-value < 0.05: *, 0.001 < *p*-value < 0.01: **, *p*-value < 0.001: ***. Colors represent the location of the measured parameters (Yellow = White Matter, Green = Gray Matter and Orange = Central canal).

The AW distances were significantly affected by sex (*p* < 0.001), showing higher values and variability for 3-weeks-old females (987.45 ± 387.36 μm) than for 3-weeks-old males (734.75 ± 151.65 μm). The PW distances did not differ significantly by sex (*p* = 0.71) but were influenced by age (*p* = 8.80e-03), and seemed to decrease in older animals. LVH values were significantly influenced by both sex (*p* = 1.71e-04) and age (*p* = 9.01e-08). The highest mean value was observed in 3-weeks-old females (543.62 ± 209.04 μm) and 3-weeks-old males (404.41 ± 154.27 μm). For the 8-weeks-old group, both male and female values decreased (female: 97.87 ± 64.81 μm; male: 93.89 ± 83.21 μm). Same observations were made for the RVH values with the highest values found for the 3-weeks-old females (547.09 ± 211.87 μm) while all 8-week-old mice showed lower values (female: 63.14 ± 61.04 μm; male: 72.90 ± 102.38 μm). Both LDH and RDH showed a strong significant effect of sex (LDH: *p* = 2.27e-07; RDH: *p* = 5.85e-06) but no significant effect of age (*p* = 0.26 and 0.62 respectively). 3-weeks-old females showed the highest LDH (700.23 ± 123.78 μm) and RDH (611.99 ± 142.90 μm) values. These values decreased significantly in 8-weeks-old females (LDH: 311.10 ± 99.54 μm; RDH: 309.47 ± 49.15 μm), while males presented less differences between age groups. This may indicate that gray matter in females is more dynamically regulated during early postnatal development, possibly due to hormonal fluctuations. The LVH-LDH ratio was significantly influenced by both sex (*p* = 7.63e-06) and age (*p* = 1.00e-04). The ratio was highest in 3-weeks-old females (1.34 ± 0.23). The RVH-RDH ratio was significantly influenced between sex (*p* = 0.03) but not age (*p* = 0.14). The VGC distance was highest in 3-weeks-old females (145.72 ± 80.12 μm) and decreased with age, particularly in this group. The DGC distance showed significant differences by sex (*p* = 2.05e-05) and age (*p* = 0.015). The VGC-DGC measurement was also significant for both sex and age (*p* < 0.001). The highest value was observed for the 3-weeks-old females (250.32 ± 108.51 μm) then decreased with age. The commissure size was significantly influenced by both sex (*p* = 2.13e-08) and age (*p* = 5.58e-13). The highest values were found in 3-weeks-old females (455.62 ± 148.24 μm) compared to 8 weeks-old females (400.10 ± 148.24 μm).

#### 3.2.2 Central canal

**Figure 6:**
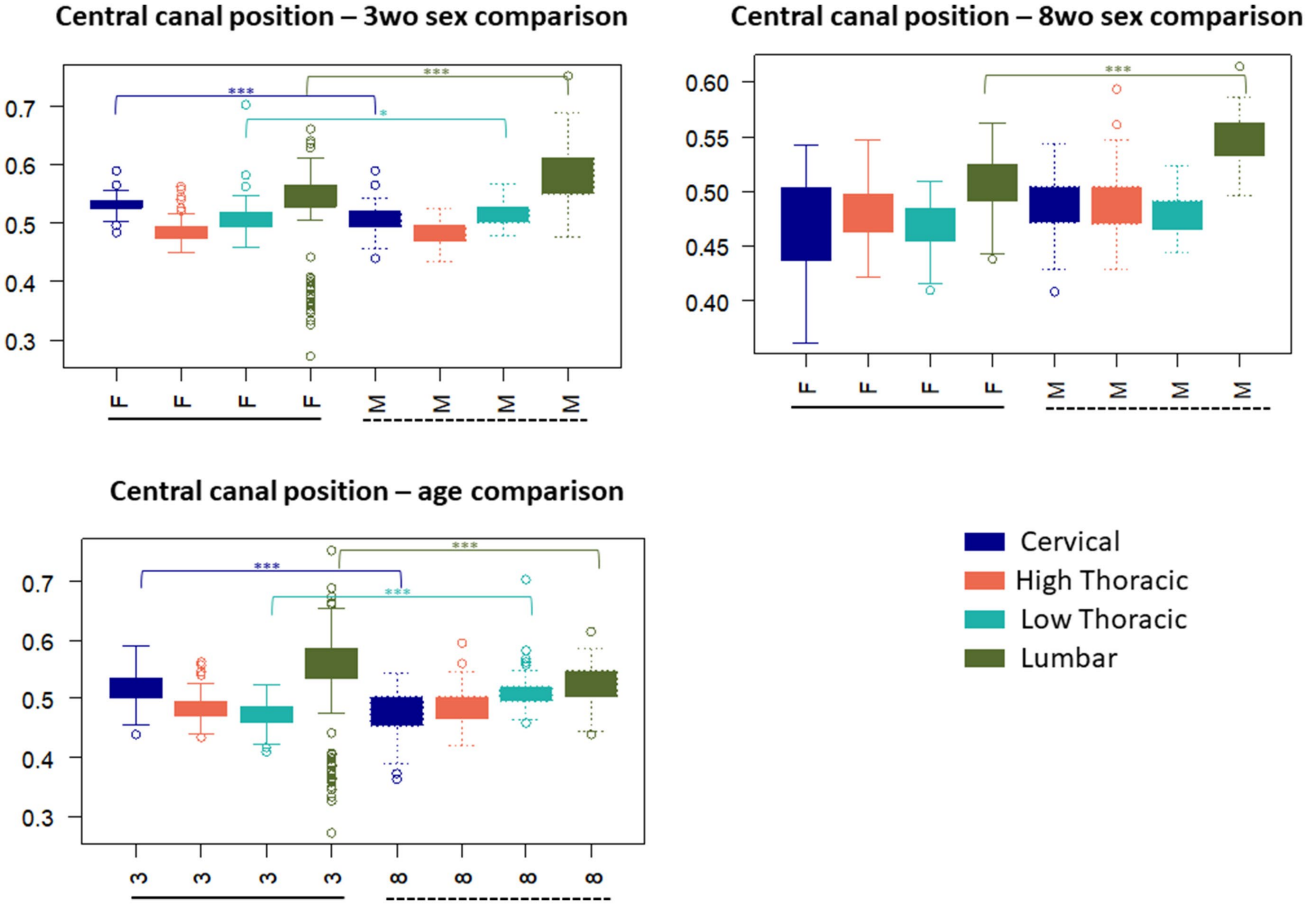
Central canal position (normalized ratio VWC (VWC + DMS)) variation according to the spinal regions and influence of sex and age on the values – 0.01 < *p*-value < 0.05: ∗, 0.001 < *p*-value < 0.01: ∗∗, *p*-value < 0.001: ∗∗∗.

The 3-weeks-old female mice exhibited a higher CC position in the cervical and lumbar regions compared to the-3 weeks-old males, with significantly different values in these same regions (p < 0.001). A lower, but significant difference was noted in the low thoracic region (*p* < 0.05). When considering all individuals regardless of sex, it seemed that the CC position became more ventral from the cervical to the high thoracic segments, before moving dorsally again in the low thoracic and lumbar segments. For the 8-weeks-old group, sex-related differences were less pronounced. A significant difference between males and females was only observed in the lumbar region (*p* < 0.001). This group also displayed less variability in the CC position compared to the 3-weeks-old group, particularly when sex was taken into account (Figure 6). The higher position of the CC in younger animals may relate to overall tissue expansion during early spinal cord development, which gradually stabilizes with maturation. Increased variability in CC position among younger and especially female animals may reflect a higher sensitivity to developmental, hormonal, or metabolic influences at early stages.

Significant differences between ages were identified in the cervical, high thoracic, and lumbar regions (*p* < 0.001). Additionally, while younger mice showed more variability. The CC position values tended to be the most dorsal in the cervical and lumbar regions. Older mice showed a more gradual increase from cervical to lumbar segments.

The CC size tended to be slightly larger in 8-weeks-old animals, with an increase of the mean ellipse area at 8-weeks-old (2199.50 ± 569.44 μm^2^) compared to 3-weeks-old (1925.58 ± 630.16 μm^2^). This difference was mainly due to an increase in both the smallest and largest diameters (Table 3). Female mice showed a wider smallest diameter than males (20.25 ± 4.87 μm and 17.62 ± 4.38 μm respectively), resulting in a slightly more circular CC shape reflected by a lower mean eccentricity (0.85 for females and 0.90 for males). Along the rostro-caudal axis, the cervical region displayed the largest CC area (2120.35 ± 549.40 μm^2^), followed by lumbar (2359.50 ± 543.94 μm^2^), low thoracic (1886.73 ± 554.15 μm^2^), and high thoracic regions (1774.52 ± 581.70 μm^2^). Along this axis, the largest diameter showed little variation, whereas the smallest diameter became narrower in the thoracic regions, which contributed to a more elliptical appearance of the CC. Accordingly, eccentricity values were lowest in cervical and low thoracic regions, and highest in the lumbar segment (0.90 ± 0.06), suggesting a more elliptical shape in the caudal spinal cord.

**Table 3:** Variations in central canal dimensions (smallest and largest parameters, ellipse area and eccentricity) and comparisons between age, sex and spinal segments.

Parameters	3wo	8wo	Female	Male
Smallest diameter (μm)	17.81 ± 4.65 [2.96; 47.2]	19.81 ± 4.73 [3.38; 37.48]	20.25 ± 4.87 [3.38; 47.2]	17.62 ± 4.38 [2.96; 37.48]
Largest diameter (μm)	42.53 ± 7.40 [4.77; 71.79]	44.43 ± 9.11 [8.15; 78.14]	41.84 ± 7.31 [10.87; 71.79]	45.03 ± 8.96 [4.77; 78.14]
Ellipse area (μm^2^)	1925.58 ± 630.16 [49.25; 7018.79]	2199.50 ± 569.44 [85.29; 4877.61]	2146.39 ± 632.91 [110.66; 7018.79]	1998.36 ± 589.85 [49.25; 4977.61]
Eccentricity	0.89 ± 0.09 [0.27; 0.99]	0.87 ± 0.10 [0.17; 0.98]	0.85 ± 0.10 [0.17; 0.99]	0.90 ± 0.08 [0.24; 0.99]

Parameters	Cervical	High thoracic	Low thoracic	Lumbar
Smallest diameter (μm)	20.84 ± 5.72 [2.96; 33.58]	17.60 ± 4.47 [3.38; 47.20]	17.72 ± 4.06 [4.06; 40.46]	19.58 ± 4.18 [9.31; 34.14]
Largest diameter (μm)	41.96 ± 12.00 [6.61; 78.14]	39.60 ± 5.85 [12.91; 64.3]	41.91 ± 6.54 [4.77; 67.09]	47.81 ± 7.03 [32.01; 72.92]
Ellipse area (μm^2^)	2120.35 ± 549.40 [49.62; 3704.84]	1774.52 ± 581.70 [110.66; 7018.79]	1886.73 ± 594.15 [49.25; 6500.19]	2359.50 ± 543.94 [1011.11; 4088.89]
Eccentricity	0.79 ± 0.15 [0.17; 0.99]	0.88 ± 0.70 [0.42; 0.98]	0.89 ± 0.07 [0.52; 0.99]	0.90 ± 0.06 [0.52; 0.99]

Data are classed according to mice age and sex. – Mean ± standard deviation [minimum value, maximum value].

Overall, when computing the respective coefficients of variation, the minor and major diameters as well as the central canal area show little variation (<15%).

### 3.3 Influencing parameters

Most of the measured lengths and computed ratios for both WM and GM were significantly influenced by sex and age (*p* < 0.001). Sex had an influence on all parameters except for the AP-⌀T ratio, which remained statistically unchanged when comparing the females and males’ groups (Table 4). Age significantly affected all distance measurements, the commissural size, the VGC–DGC distance and the LVH-LDH ratio (*p* < 0.001). Some ratios such as AP-⌀T and RVH-RDH were less influenced by age (*p* < 0.05), while the AW-PW ratio did not show a significant effect of age. In comparison, the spinal regions had more impact on the spinal cord anatomy. While distances such as ⌀T, DMS, LWM, AW, and PW were significantly influenced by the considered spinal regions (*p* < 0.001), the others parameters remained unaffected. All computed ratios were significantly different along the rostro-caudal axis (*p* < 0.01), with the exception of VGC–DGC, whose values remained similar across spinal regions (Table 4).

**Table 4:** Influence of age, sex and spinal regions (SR) on morphological parameters.

Length (μm)		*p*-value age	*p*-value sex	*F* value VL	*p*-value SR	*F* value MANOVA	*p*-value MANOVA
White matter	AP	<2e-16 ***	<2e-16 ***	0.55	0.46		
	T	< 2.2e-16 ***	< 2.2e-16 ***	10.03	1.60e-03**	14.10	1.84e-04 ***
	VWC	<2e-16 ***	<2e-16 ***	1.58	0.21		
	DMS	9.03e-12 ***	< 2.2e-16 ***	55.72	1.88e-13 ***	80.07	< 2.2e-16 ***
Gray matter	LWM	< 2.2e-16 ***	< 2.2e-16 ***	11.05	9.00e-04 ***	0.02	0.89
	MWM	< 2.2e-16 ***	< 2.2e-16 ***	10.18	1.50e-03 **	3.30	0.07
	AW	< 2.2e-16 ***	< 2.2e-16 ***	29.53	7e-08 ***	10.89	1.00e-03 **
	PW	7.24e-15 ***	4.32e-06 ***	5.71	0.02*	45.98	2.11e-11 ***
	LVH	< 2e-16 ***	< 2e-16 ***	4.85	0.03*	31.20	3.04e-08 ***
	RVH	< 2.2e-16 ***	< 2.2e-16 ***	6.96	8.e-03 **	2.51	0.11
	LDH	<2e-16 ***	<2e-16 ***	1.15	0.28		
	RDH	<2e-16 ***	<2e-16 ***	0.48	0.49		
Central canal	VGC	< 2.2e-16 ***	< 2.2e-16 ***	13.65	2.e-04 ***	220.34	< 2.2e-16 ***
	DGC	< 2.2e-16 ***	1.8e-12 ***	14.24	2.e-04 ***	2.52	0.11
Ratio							
White matter	AP/T	1.06e-02 *	0.26	22.25	2.75e-06	30.90	3.53e-08 ***
	Commiss_size	7.69e-16 ***	< 2.2e-16 ***	7.68	5.30e-03 **	0.15	0.70
Gray matter	AW/PW	0.16	<2e-16	112.48	<2e-16	177.62	< 2.2e-16 ***
	RVH/RDH	4.00e-04**	1.35e-05 ***	42.66	1.06e-10 ***	0.24	0.62
	LVH/LDH	1.84e-12 ***	5.13e-15 ***	53.08	6.71e-13 ***	0.84	0.36
Central canal	VGC + DGC	<2e-16 ***	<2e-16 ***	0.33	0.56	4.84	2.81e-02 *
Age						71.09	< 2.2e-16 ***
Sex						3.48	0.06

Multivariance analysis is used (0.01 < *p*-value < 0.05: ∗, 0.001 < *p*-value < 0.01: ∗∗, *p*-value < 0.001: ∗∗∗). Data are classed according to measured morphological parameters and the computed ratios. Green boxes mean statistically significant values.

### 3.4 Influence of spinal regions

VGC and DMS distances were the most influenced by the spinal regions, with *F*-values of 220.24 and 80.07, respectively, (Table 4). Other measurements such as AW, PW, LVH and ⌀T also showed significant differences between spinal segments, with F-values ranging from 10.89 to 45.98. The remaining parameters did not show notable variation. Regarding the computed ratios, AW-PW (*F* = 177.62) and AP-⌀T (*F* = 30.90) were the most strongly influenced by spinal cord regions. These results indicate that while spinal region has a significantly impact, age remains the most influential factor as reflected by a *F*-value of 71.09 (Table 4).

### 3.5 Correlations between morphological parameters

From the correlation matrix presented in Figure 4B a strong correlation was found between parameters linked with the transverse diameter: ⌀T, MWM and LWM (*r* = 0.93). MWM and LWM were also logically correlated (*r* = 0.83). The length AW was strongly correlated with parameters linked with the transverse diameter (⌀T, LWM, MWM, *r* = 0.78) and with ventral (LVH *r* = 0.79, RVH *r* = 0.80) and dorsal horns lengths (LDH *r* = 0.67, RDH *r* = 0.66). The distance VWC was strongly correlated with AW (*r* = 0.64), with ventral (LVH *r* = 0.72, RVH *r* = 0.70) and dorsal horns lengths (LDH, RDH *r* = 0.69) and with the transverse diameter parameters (⌀T *r* = 0.70, LWM, MWM, *r* = 0.79). The distance AP is correlated with VWC (*r* = 0.81), AW (*r* = 0.71), the transverse diameter parameters (⌀T *r* = 0.74, LWM, MWM, *r* = 0.73), the ventral (LVH, RVH *r* = 0.74) and dorsal horns lengths (LDH, RDH *r* = 0.70) and DMS (*r* = 0.66). Ventral and dorsal horns lengths are highly correlated with all parameters (*r* > 0.5) except the central canal related ones (DGC and VGC).

### 3.6 PKD2L1^+^ cells

The distribution of PKD2L1^+^ cells along the spinal cord axis and as a function of the animal age and sex was assessed using the selective expression of tdTomato. Here our aim was to analyze the variation in number and distance of the so-called CSF-cNs present around the CC. Nevertheless, in agreement with previous studies (Jurčić et al., 2021; Gerstmann et al., 2022; Nakamura et al., 2023), we also found tdTomato (i.e., PKD2L1^+^) cells further away from the CC especially in older animals. We therefore distinguished in our study these two populations. In the rest of our study, CSF-cNs with the characteristic morphology and clearly in contact with the CSF are referred as proximal while cells further away are named distal (see below). Figure 3A illustrate a representative spinal cord section used to carried out the analysis of PKD1L1^+^ cells visualized from tdTomato expression (red; the CC was localized using DAPI staining, blue). The quantification of PKD2L1^+^ cells or CSF-cNs per histological slice (thicknesses = 27.07 ± 7.92 μm) revealed few variations between age groups, sexes, and spinal regions. At 3-weeks, the average number of cells per slice was 7.88 ± 3.40, increasing slightly to 9.06 ± 5.92 at 8 weeks. Females showed a lower number of cells (6.82 ± 3.50) compared to males (10.00 ± 5.71) and significant effect of sex was assessed with a Wilcoxon Mann–Whitney test (*p* = 6.94e-07). Regarding spinal regions, the number of cells per slice ranged from 7.32 ± 3.64 in the cervical region to 9.32 ± 4.75 in the lumbar region, with intermediate values observed in thoracic segments (Table 5). These data are in agreement with data recently reported (Crozat et al., 2025).

**Table 5:** Quantification of PKD2L1+ cells’ number per slices and computation of the distance between their centroid and the CC center previously computed.

Parameters	3wo	8wo	Female	Male
Number of cells/slices	7.88 ± 3.40 [2; 16]	9.06 ± 5.92 [1; 32]	6.82 ± 3.50 [1; 21]	10 ± 5.71 [2; 32]
Distance from CC center (μm)	83.83 ± 50.55 [2.8; 214]	96.08 ± 52.98 [0.63; 226.83]	84.40 ± 52.12 [6.82; 226.1]	95.53 ± 52.21 [0.63; 226.83]

Parameters	Cervical	High thoracic	Low thoracic	Lumbar
Number of cells/slices	7.32 ± 3.64 [2; 17]	8.3 ± 4.63 [2; 27]	9.12 ± 6.42 [1; 32]	9.32 ± 4.75 [2; 21]
Distance from CC center (μm)	85.13 ± 50.80 [5.37; 209.86]	90.17 ± 51.27 [2.8; 214]	93.94 ± 53.20 [3.73; 226.16]	94.51 ± 53.17 [0.63; 226.83]

Comparisons between age and sex. Data are classed according to mice age, sex and spinal segments. – Mean ± standard deviation [minimum value, maximum value].

The average distance of cells centroid from the CC center also showed few differences. PKD2L1^+^ cells were located at 83.83 ± 50.55 μm for the 3-weeks-old group and 96.08 ± 52.98 μm for the 8-weeks-old group. Females showed cells at a distance of 84.40 ± 52.12 μm, whereas males had cells at 95.53 ± 52.21 μm. Spinal regions analysis showed distances ranging between 85.13 ± 50.80 μm (cervical) and 94.51 ± 53.17 μm (lumbar).

It was observed that the main population of tdTomato^+^ cells were located proximally (< 40 μm) with a smaller number of cells at more distal positions. While the proximal cells likely represent CSF-cNs, the identity of the more distant tdTomato^+^ cells are uncertain (but see Nakamura et al., 2023). Therefore, our analysis focuses on spatial distribution patterns and does not assume functional equivalence between proximal (up to 100 μm from the CC center) and distal (from 100 to 230 μm from the CC center) subgroups (Figure 3B). The total number of PKD2L1+ cells distributed according to their distance from the CC and across different spinal cord regions for the two age groups (3 and 8 weeks) is presented in Figure 3C. The lower thoracic and lumbar segments showed the highest number of cells, with both regions showing more than 200 cells at 8 weeks. Regardless of the segment, the majority of PKD2L1+ cells (i.e., CSF-cNs) were located within the 0–100 μm range from the CC center for both age groups (Figure 3).

Figure 3D further illustrates this regional and age-related variation showing the percentage of PKD2L1^+^ cells within each distance category. In the cervical region, at 3 weeks, cells were evenly located within the proximal zone (50%) and the distal zone (50%). By 8 weeks, this proportion shifted, with 63% of cells remaining close to the CC and only 37% found in the distal area. For the other spinal segments, an opposite trend was observed. In the high thoracic region, 61.5% of cells at 3-weeks-old and 50.2% at 8-weeks-old were located proximally, while 38.5 and 48.8%, respectively were found more distally from the CC center. The increase of cells in the distal region with age is also observed in the low thoracic (38.3% and 52.4% for the 3-weeks-old and 8-weeks-old group respectively) and lumbar segments (37.8 and 54% for the 3-weeks-old and 8-weeks-old group respectively). As for the cells close to the CC in these regions, their percentage decrease with age.

**Figure 7:**
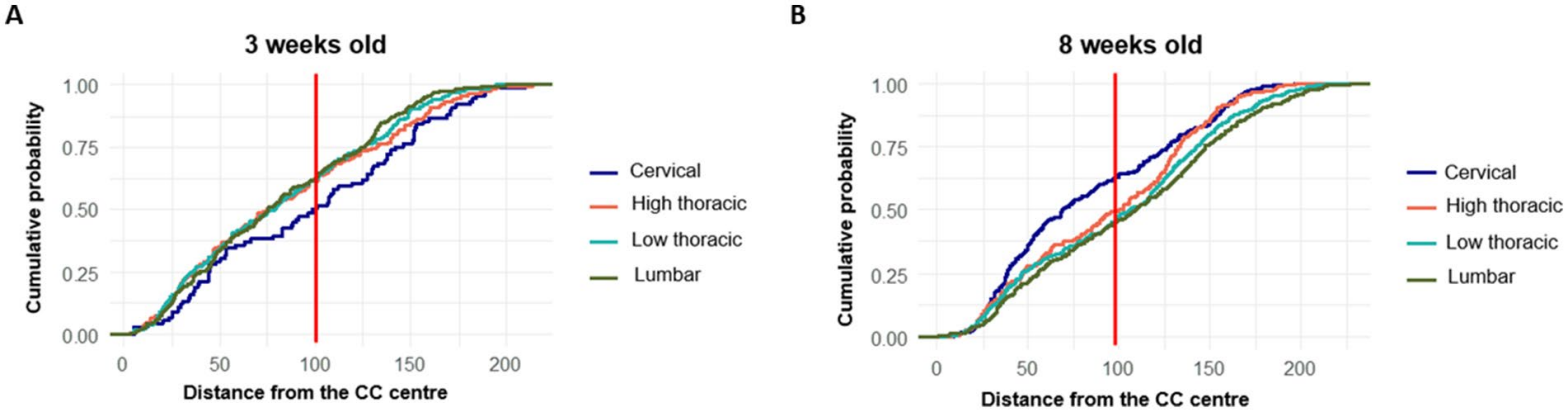
Cumulative distribution of PKD2L1+ cells according to their distance from the central canal center across spinal segments and according to the age **(A)** 3-weeks-old mice and **(B)** Distribution in 8-weeks-old mice.

The cumulative probability distributions of PKD2L1^+^ cell distances from the CC center were plotted for the different spinal regions at 3 (Figure 7A) and 8 weeks (Figure 7B) to confirm the previous observations. In 3-weeks-old animals, the cervical region displayed a lower number of neurons near the CC, with a higher proportion located farther from the center compared to other segments. In contrast, the high thoracic, low thoracic, and lumbar segments showed steeper curves, suggesting that a larger proportion of neurons were concentrated closer to the CC, particularly in high and low thoracic and lumbar regions. By 8 weeks, a noticeable shift occurred in the cervical curve, which became steeper, indicating that more neurons were now found near the CC, while the curves for caudal regions flattened slightly.

Although some differences were found between age and spinal segments, the performed statistical analysis only showed some trend but were not conclusive due to the high cells distribution variability between the spinal regions.

## 4 Discussion

This study provides a comprehensive anatomical characterization of the mouse spinal cord while analyzing the effect of age, sex and spinal regions. Age emerged as the strongest factor, driving progressive structural reorganization of GM, WM, and the CC, while sex exerted more Suttle but still detectable effects, particularly in young animals. In addition, spinal region strongly shaped local morphology along the rostro-caudal axis, and PKD2L1^+^ cells, the CSF-cNs, consistently clustered around the central canal, with clear regional and age-related variations.

### 4.1 Influence of age

As expected, age appeared to be a major influencing factor on spinal cord morphometry. Overall, greater variability was observed in the measured distances for the 3-weeks-old group compared to the 8-weeks-old group. This variability reflects the anatomical heterogeneity characteristic to postnatal development, during which growth processes are progressing and are not fully synchronized among individuals. The observed decrease in anteroposterior (AP) length between the 3 and 8-weeks-old groups may indicate progressive morphological adjustments of the spinal cord, possibly reflecting tissue compaction during growth. In contrast, transverse diameters (⌀T) remained relatively stable, suggesting that lateral expansion of the spinal cord may occur earlier and appears to stabilize before the ventro-dorsal modifications are complete. The ventral commissure deepens with age and the GM arrangement become more and more narrow around the CC, particularly between the ventral and dorsal gray commissures (VGC and DGC). These findings suggest ongoing postnatal maturation of the central region of the spinal cord, possibly reflecting continued development of left–right connectivity, ensuring sensory processing and motor function (Maxwell and Soteropoulos, 2020). This also may suggest changes in neuronal structure and maturation (Fu et al., 2015). The WM architecture also changes with age. While AW and PW values evolved differently with age, the increase of the AW-PW ratio in older animals which could reflect continued development of commissural pathways or local microcircuitry, in line with previous studies. GM morphology was also impacted. The lengths of dorsal and ventral horns (LVH, RVH, LDH, RDH) were reduced in older animals, which may indicate structural reorganization within GM laminae possibly due to neuronal cells migration. Regarding age-dependent changes of the CC, its position varied along the rostro-caudal axis and differed significantly between age groups, particularly in the cervical and lumbar regions. This suggests that growth does not occur uniformly along the spinal cord and may affect the spinal cord enlargement. Additionally, the CC area tended to increase with age, mainly due to a slight enlargement of both the smallest and largest diameters. The overall shape also appeared more regular, as reflected by lower eccentricity values, and may be influenced by developmental processes affecting canal geometry and surrounding tissues. These observations suggest that the mouse spinal cord undergoes progressive morphological changes between 3-weeks-old and 8-weeks-old.

### 4.2 Influence of sex

Sex contributes to variations in spinal cord structure, but age appears to be the more dominant factor (Laing et al., 2014). 3-weeks-old females exhibited higher mean values and greater variability across numerous measurements, including AP, DMS, AW and both dorsal and ventral horn lengths (LVH, RVH, LDH, RDH). These observations suggest a larger anatomical heterogeneity in young females, which may reflect a slightly earlier developmental stage compared to young males. Sex also influenced some computed ratios such as LVH-LDH and RVH-RDH, indicating subtle differences in GM organization between males and females. As for the CC, it appeared to be wider in females.

### 4.3 Influence of spinal regions

The shape of the WM and GM evolves along the rostro-caudal axis, reflecting regional adaptations of the spinal cord morphology. In the cervical region, the transverse diameter of the spinal cord is generally wider, and the ventral and dorsal horns are more developed, giving the GM a butterfly-like appearance with well-defined lateral expansions. Toward the thoracic region, both the GM and WM undergo changes. The GM appears more centered within the spinal cord, with narrower dorsal and ventral horns. The WM also becomes proportionally more dominant in the high thoracic region. In the lumbar region, the GM expands again, particularly for the ventral horns, showing its widened morphology. The enlargements observed at the cervical and lumbar segments may reflect the higher density of motoneurons in the ventral horn that are required for fore- and hindlimb muscle control for locomotion (Kuehn et al., 2023; Moshonkina, 2008). The WM remains present but with a slightly reduced cross-sectional area compared to the highest regions. Regarding the CC, its position and shape were not uniformly distributed along the spinal cord. The position of the CC followed a non-linear rostro-caudal pattern, with more ventral values observed in the high thoracic region and higher positions in cervical and lumbar segments. Additionally, its morphology changed along the axis, becoming more elliptical in the thoracic regions, as reflected by increased eccentricity values. These observations highlight a clear adaptation of the spinal cord morphology along its rostro-caudal axis. The shape of GM and WM adapt according to functional demands leading to a reduction in space in the thoracic region.

### 4.4 PKD2L1^+^ cells distribution

This study also provides a quantitative analysis of the distribution of PKD2L1^+^ cells along the mouse spinal cord, focusing on their proximity to the CC across the spinal segments. It is known that in Pkd2l1-Cre::tdTomato mice, in particular older than 4 weeks, cell types more distant from the CC and without the characteristic morphology of CSF-cNs can be observed (Jurčić et al., 2021; Gerstmann et al., 2022; Nakamura et al., 2023). However, Nakamura et al. (2023) indicated that some of the distal Pkd2l1^+^/tdTomato^+^ cells would represent oligodendrocytes (see Figures 3E,G in their study) that were shown to express Pkd2l1 mRNA (Russ et al., 2021). Further, distal PKD2L1^+^ cells, at least those present along the ventral midline, exhibit electrophysiological properties similar to those of CSF-cNs are Jurčić et al. (2021). Finaly, Gerstmann and colleagues (Gerstmann et al., 2022) indicated that in the Pkd2l1-Cre::eGFP mouse model over 80% of the eGFP expressing cells were also PKD2L1^+^. Therefore, rather than considering possible Cre-recombinase leakage, these data suggest the presence of cells that express or were produced from percussors expressing Pkd2l1 whose developmental origin needs to be further investigated. Moreover, the absence, to date, of electrophysiological or connectivity validation further limits our ability to attribute functional identity to all tdTomato^+^ cells. These aspects should be addressed in future work using complementary approaches. We therefore mainly focused in our study on cells in the proximal region with the characteristic morphology and dendritic projections observed in the CC (Crozat et al., 2025). The results reveal nevertheless a constant spatial organization of PKD2L1^+^ cells, with a higher density located within 100 μm from the CC center across all spinal regions. The lumbar and lower thoracic regions demonstrated the highest number of PKD2L1^+^ cells. This rostro-caudal distribution may correspond to regional differences in development or architecture of CC-adjacent cells. Further the tendency of PKD2L1^+^ neurons to cluster around the CC agrees with their roles, as CSF-cNs, in sensing changes in the CSF environment (Jalalvand et al., 2016, 2018; Knafo and Wyart, 2018). The lumbar region is known to contain locomotor circuits, where motoneurons controlling hindlimb muscles are present (Zhang et al., 2012). This could explain the highest distribution of PKD2L1^+^ cells in this region as supported by the motor alterations observed in animals where CSF-cNs were genetically ablated or inhibited (Gerstmann et al., 2022; Nakamura et al., 2023). The increased number of cells in caudal regions may support such a function for CSF-cNs but further functional validation is necessary and need to be extended to the other spinal cord segments. However, while the overall number of PKD2L1^+^ cells increased in the caudal regions, the proportion of cells and thus neurons located within the close CC area decreased slightly at the low thoracic and lumbar segments when compared to the farthest area. This may reflect a spatial shift in neuronal distribution. Alternative explanations such as anatomical growth or marker expression dynamics cannot be excluded as well as developmental growth dynamics or differential labeling. Indeed, this increase may reflect regional differences in anatomical organization, although functional significance remains to be established, and the development of neuronal networks of PKD2L1^+^ cells in the lower segments of the spinal cord (Crozat et al., 2025). The presented results align with other studies that analyzed the CSF-cNs positions along the spinal cord (Crozat et al., 2025; Nakamura et al., 2023).

It is important to note that the increase in neurons between the two age groups may be due to an enhanced differentiation of PKD2L1-positive cells between 3 and 8 weeks (Gerstmann et al., 2022; Nakamura et al., 2023), explaining the increase of cells in the distal regions. Indeed, the Pkd2l1-Cre driven expression of tdTomato does not allow strict discrimination between CSF-cNs and other cellular populations. It is known that in Pkd2l1-Cre::tdTomato mouse cell types other than CSF-cNs can be labeled (Nakamura et al., 2023). However, the spatial patterns observed, such as changes in average distance from the CC across age or sex, remain biologically informative. These trends are interpreted as potential indicators of anatomical growth rather than definitive changes in specific cell types. The full population of tdTomato-positive cells is therefore analyzed as a centrally localized population of PKD2L1^+^ cells surrounding the CC.

### 4.5 Limitations

This work has several limitations. First, a larger sample size could have enhanced the statistical power and further validate the robustness of the observed findings. Nevertheless, the results based on our statistical analysis indicate that the sample size is discriminative enough to draw reliable conclusion; Moreover, the sample size used allow to reconcile sufficient statistical power with ethical issue when using animal models (the 3R rules). Although 811 sections were analyzed, they do not represent independent biological replicates, as the true replicate is the animal (*n* = 18). Treating sections as statistical units allowed the assessment of intra-animal variability, but results should be interpreted in light of this design. The large number of morphometric parameters increases the risk of inflated significance; given the exploratory nature of the work, findings should be considered as hypothesis-generating and require confirmation in larger cohorts. Second, the use of PFA fixation, slicing protocols and tissue manipulation during microscope slides mounting introduce distortions due to tissue shrinkage or deformation, especially when studying the CC shape. Indeed, many outliers were found when focusing on the eccentricity. These eccentricity values vary between 0.15 and 0.60, suggesting that the ellipsoidal shape may not be physiological but may be induced by the slicing procedure. Although uniform processing minimized such effects, future studies either *in-vivo* or without fixation procedure using MRI imaging methods could be an alternative providing a sufficient resolution can be achieved with this method. The counting of PKD2L1^+^ cells was also affected by the 24 h PFA fixation as it decreases the tdTomato fluorescence. When close to each other, the algorithm was not capable of distinguishing all neurons separately, causing an underestimation of cells number. Although the image analysis method enabled high-throughput analysis across hundreds of sections, it does not fully correct for potential biases introduced by section thickness or object overlap. Also, the variability in the slices’ thicknesses (27.07 ± 7.92 μm) leads to an inhomogeneous distribution of the PKD2L1^+^ cells number across the spinal segments and less statistical power. Third, because user-friendly registration tools for spinal cord sections are not available, manual landmarking was used as the most realistic approach, while the development of a deep-learning model remained out of scope. In addition, although independent slice normalization combined with a global threshold and size filter reduced bias, variability in segmentation due to staining intensity or tissue quality cannot be fully excluded. Further validation, requiring electrophysiology or connectivity mapping protocols, is necessary to better explain the neurons distribution and to support functional interpretations.

## 5 Conclusion

This study offers a comprehensive morphometric dataset of the mouse spinal cord, revealing how age, sex, and rostro-caudal location shape gray and white matter architecture and central canal morphology. We demonstrate that age is the dominant factor influencing structural variability, with clear postnatal changes in ventral and dorsal horn dimensions, commissural depth, and central canal geometry. Sex-related differences are more subtle but highlight increased anatomical variability in young females. Importantly, this study provides a quantitative mapping of PKD2L1^+^ cells distribution, showing their preferential localization near the central canal and their increased density in lumbar segments, potentially reflecting regional specialization. By combining high-resolution anatomical data with spatial neuron mapping, these findings supply a valuable resource for spinal cord modeling and developmental anatomy. The integration of age- and sex-specific features can enhance the realism of computational models and provide a framework for future studies investigating cerebrospinal fluid-contacting neurons.

## Data Availability

The raw data supporting the conclusions of this article will be made available by the authors, without undue reservation.
