# Supplementary material for: Spinal cord morphology and PKD2L1+ cells distribution: effects of age, sex, and spinal segment in mice
Source: Front Neuroanat. 2025 Oct 24;19:1652848. doi: 10.3389/fnana.2025.1652848 (PMC12592075; doi:10.3389/fnana.2025.1652848)
Supplement: Supplementary file 1 [file Data_Sheet_1.pdf]

Supplementary data

Table 1 – Summary of Sample and Slices Distribution by Age, Sex, Spinal Segment, and Genotype

**3 weeks old**				**8 weeks old**			
**Mice**		**Weight (g)**	**Type**	**Mice**	**Sex**	**Weight (g)**	**Type**
1	Female	15	Wild Type	10	Female	14	Wild Type
2	Female	15	Wild Type	11	Female	17	Wild Type
3	Female	7	Wild Type	12	Female	18	PKD2L1 +
4	Female	7,6	PKD2L1 +	13	Female	18	PKD2L1 +
5	Female	5,5	PKD2L1 +	14	Male	19	PKD2L1 +
6	Male	9,2	Wild Type	15	Male	22	Wild Type
7	Male	4,7	PKD2L1 +	16	Male	23	PKD2L1 +
8	Male	6,2	Wild Type	17	Male	18	Wild Type
9	Male	6,4	PKD2L1 +	18	Male	19	Wild Type
**Sex**	**Spinal segments**		**Nbr of samples**	**Sex**	**Spinal segments**		**Nbr of samples**
Female	Cervical		2	Female	Cervical		3
	High thoracic		3		High thoracic		3
	Low Thoracic		3		Low Thoracic		4
	Lumbar		3		Lumbar		2
Male	Cervical		2	Male	Cervical		3
	High thoracic		2		High thoracic		3
	Low Thoracic		3		Low Thoracic		3
	Lumbar		4		Lumbar		2

**Slices**	**N**
**Total**	**811**
Female_3_wo	202
Male_3_wo	199
Female_8_wo	211
Male_8_wo	199
