# Supplementary material for: Spinal cord morphology and PKD2L1+ cells distribution: effects of age, sex, and spinal segment in mice
Source: Front Neuroanat. 2025 Oct 24;19:1652848. doi: 10.3389/fnana.2025.1652848 (PMC12592075; doi:10.3389/fnana.2025.1652848)
Supplement: Supplementary file 2 [file Data_Sheet_2.pdf]

Supplementary data

Table 2 – Influence of genotype on morphological parameters. Linear models including genotype, age, and sex as fixed effects are used. The genotype effect is assessed using ANOVA and the p-values are reported as followed: 0.01< p-value < 0.05: ∗, 0.001< p-value < 0.01: ∗∗, p-value < 0.001: ∗∗∗. Data are classed according to measured morphological parameters.

**Length**		**p-value genotype**	**F_value genotype**
**WHITE MATTER**	AP	0.14	2.40
	T	0.33	1.00
	VWC	0.11	2.86
	DMS	0.14	2.46
**GRAY MATTER**	LWM	0.34	0.99
	MWM	0.31	1.09
	AW	0.23	1.56
	PW	0.40	0.75
	LVH	0.15	2.31
	RVH	0.16	2.14
	LDH	0.40	0.76
	RDH	0.40	0.77
**CENTRAL CANAL**	VGC	0.15	2.31
	DGC	0.73	0.12
